# Probiotic Supplementation Suppresses Tumor Growth in an Experimental Colorectal Cancer Liver Metastasis Model

**DOI:** 10.3390/ijms23147674

**Published:** 2022-07-12

**Authors:** Matas Jakubauskas, Lina Jakubauskiene, Bettina Leber, Angela Horvath, Kestutis Strupas, Philipp Stiegler, Peter Schemmer

**Affiliations:** 1 General, Visceral and Transplant Surgery, Department of Surgery, Medical University of Graz, Auenbruggerplatz 2, 8036 Graz, Austria; matasjakub@gmail.com (M.J.); morozovaite.lina@gmail.com (L.J.); bettina.leber@medunigraz.at (B.L.); peter.schemmer@medunigraz.at (P.S.); 2 Faculty of Medicine, Vilnius University, M. K. Ciurlionio Str. 21, 03101 Vilnius, Lithuania; kestutis.strupas@santa.lt; 3 Division of Gastroenterology and Hepatology, Medical University of Graz, Auenbruggerplatz 2, 8036 Graz, Austria; angela.horvath@medunigraz.at

**Keywords:** probiotics, colorectal cancer, liver metastasis, tumor suppression

## Abstract

Colorectal cancer (CRC) ranks third in incidence and second in mortality of all cancers worldwide. At the time of primary diagnosis, around 20% of patients already have metastatic CRC and only around 20% are candidates for radical resection. Thus, most of the patients have to undergo chemotherapy (CTx). Due to chemoresistance and side effects, novel treatment additives are crucial for controlling the disease and prolonging patient survival. The aim of this study was to evaluate probiotic supplementation and its antitumorigenic effects in an experimental CRC liver metastasis model. Six-week-old male Wistar rats received either a multispecies probiotic (1.2 × 10^9^ CFU/daily) or placebo mixture. On day 14 of the experiment, rat CRC cells (CC531) were implanted under the liver capsule later treated by FOLFOX CTx. Change in tumor volume was measured by performing micro computed tomography (micro-CT) scanning on experimental days 28 and 34. Additionally, immunohistochemical staining with anti-MPO, anti-Ki67, and anti-CD31 were performed. Tumor apoptosis was evaluated using TUNEL staining. Micro-CT image analysis indicates that probiotic supplementation significantly inhibits tumor growth. No synergistic effects between probiotic supplementation and FOLFOX CTx was observed. Reduced tumor volume was achieved by inhibiting angiogenesis, as tumor microvascular density was significantly lower in rats receiving probiotic supplementation. This study shows that a multispecies probiotic mixture significantly reduces angiogenesis and inhibits CRC liver metastasis growth in an experimental rat model.

## 1. Introduction

According to the 2020 global cancer statistics, colorectal cancer (CRC) ranks third in incidence and second in mortality of all cancers worldwide [1]. The increasing incidence in developing countries is mainly attributed to the adoption of Western-style diet and lifestyle [2]. Overall, the main risk factors for CRC include smoking, increased alcohol intake, obesity, male sex, diabetes, and inflammatory bowel disease [3]. Although the overall incidence and mortality of CRC decreased in prosperous countries due to established screening programs and improved treatment options, the problem remains relevant as the incidence of CRC among young adults (<50 years old) is rising [4,5].

At the time of primary diagnosis, around 20% of patients already have metastatic CRC and up to 50% of the patients initially diagnosed with non-metastatic CRC will develop it during the course of the disease [6,7,8,9]. Metastatic CRC has very poor outcomes, with a reported five-year survival of less than 20% [8]. The primary site of metastasis for CRC is the liver and the only possibility for a long-term cure involves the complete surgical removal of metastatic foci [6,7,10]. Unfortunately, only around 20% of such patients are candidates for radical resection, while leaving a sufficient liver volume, upon the diagnosis of CRC liver metastasis (CRCLM) [11]. In some cases, for the remaining 80% of these patients, aggressive chemotherapy (CTx) can downsize the metastasis, making them resectable.

Currently, first-line CTx consists of either oxaliplatin-based regimens (FOLFOX or CAPOX) or irinotecan-based regimens (FOLFIRI or CAPIRI). Furthermore, targeted therapy, such as vascular endothelial growth factor inhibitors, may be administered alongside [8]. CTx is regularly accompanied by many side effects and it is not well tolerated by frail patients [12,13]. Moreover, CRC is well known to develop resistance to chemotherapeutical agents, thus further promoting disease progression [14]. The main resistance development mechanisms include limitation of drug transport within the tumor cells, dysregulation of cellular physiology, and reduction of drug sensitivity through epigenetic modifications or dysregulation of miRNA levels [15]. Taking all these factors into account, novel treatment additives are crucial for controlling the disease and prolonging patient survival.

According to the WHO and expert consensus definition probiotics are “non-pathogenic live microorganisms that, when administered in adequate amounts, confer a health benefit on the host” [16]. They were shown to be beneficial in a lot of clinical scenarios, mostly associated with gastrointestinal diseases [17]. However, current research indicates that their action is widespread in the host organism and not limited to their residing place [18]. Presently, the role of probiotics in cancer treatment is gaining an increasing amount of attention. Probiotic supplementation showed promising effects in suppressing tumor progression in several animal models [19]. Unfortunately, currently, there are no animal or human studies examining the role of probiotics in treating colorectal cancer liver metastasis. The aim of this study was to evaluate probiotic supplementation antitumorigenic effects in a CRCLM rat model.

## 2. Results

### 2.1. General Data and Blood Counts

During the experiment one premature death due to CTx induced toxicity (Table 1) occurred in the Probiotics + FOLFOX Group. No other serious adverse events were noted.

**Figure 1:**
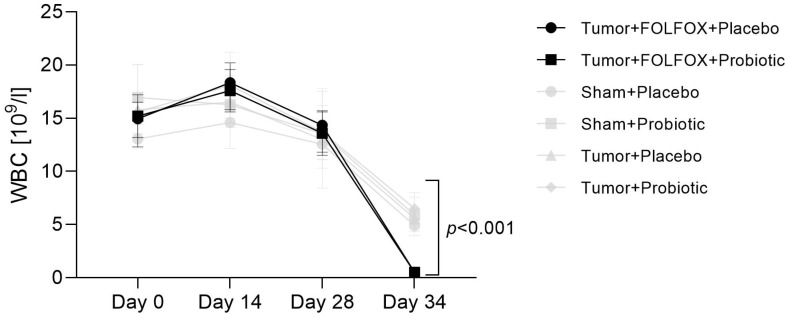
White blood cell count change throughout the study.

A single cycle of FOLFOX CTx induced severe myelosuppression as white blood cell (WBC) levels were significantly reduced in both CTx receiving rat groups when compared to rats that did not receive CTx (Figure 1). Probiotic supplementation had neither any effect on the WBC count throughout the study nor impact on the severity of leukopenia.

**Figure 2:**
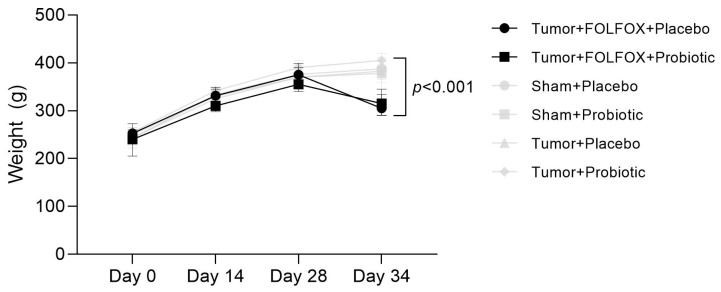
Weight change throughout the study.

Until day 28, the study rats across all groups gained weight evenly. Weight continued to grow for rats that did not receive FOLFOX CTx, however, rats that received CTx lost weight significantly (Figure 2).

### 2.2. Tumor Analysis

**Figure 3:**
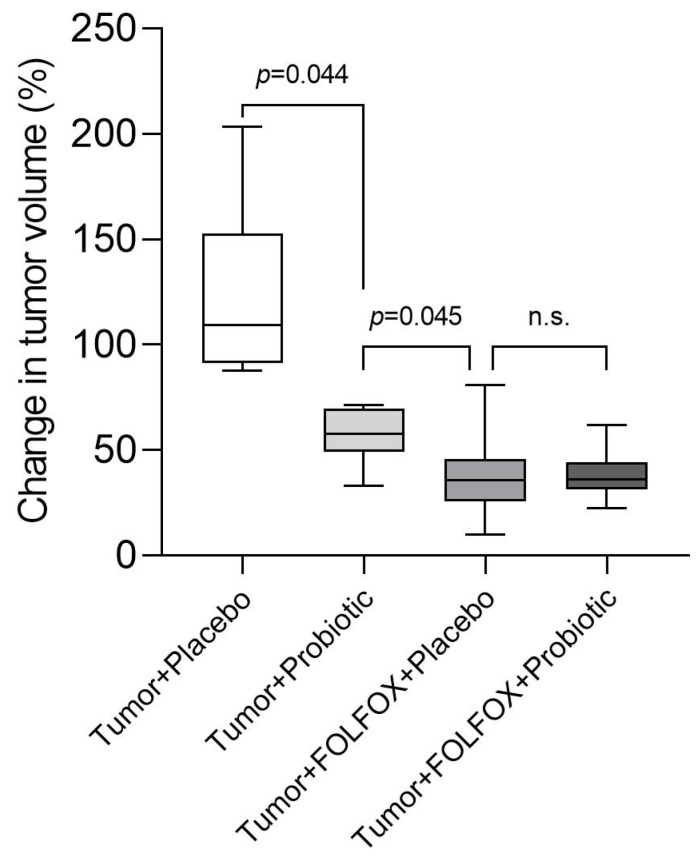
Change in tumor volume at the end of the study calculated by analyzing micro-CT images. Values outlying ± 2SD range excluded from analysis. n.s.—not significant.

Micro-computed tomography (micro-CT) image analysis indicates that probiotic supplementation alone, without FOLFOX CTx, significantly inhibits tumor growth in rats compared to the placebo group (109.20% (91.27; 152.70) vs. 57.67% (49.06; 69.76); *p* = 0.044) (Figure 3). However, there was no difference in tumor growth between intervention groups in rats that received FOLFOX CTx (35.74% (25.87; 45.65) vs. 34.90% (27.68; 43.15); *p* = 1.000).

**Figure 4:**
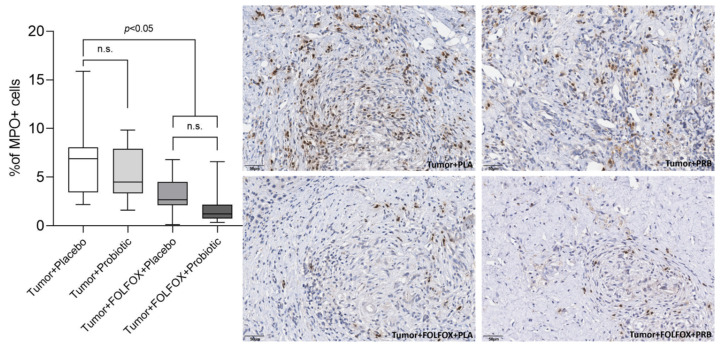
Percentage of MPO+ cells in the tumor. Values outlying ± 2SD range excluded from analysis. n.s.—not significant.

The highest percentage of MPO positive cells was observed in the tumor + placebo group. The tumor + probiotics group had a lower percentage of MPO positive cells; however, the difference was not significant (Figure 4). Both CTx groups had a significantly lower tumor infiltration with MPO positive cells when compared to both groups that did not receive CTx.

**Figure 5:**
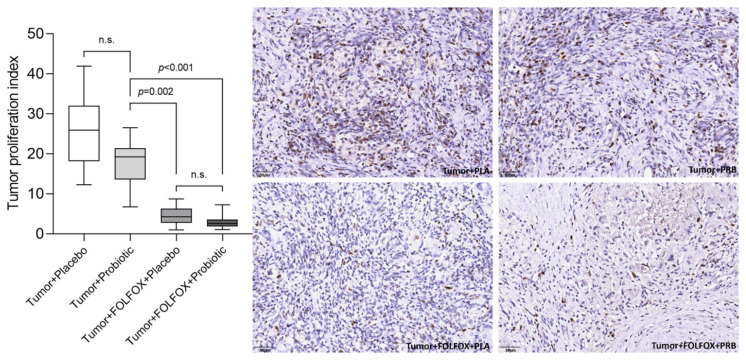
Tumor proliferation index (% of positive Ki67+ cells in the tumor). Values outlying ± 2SD range excluded from analysis. n.s.—not significant.

A similar tendency was observed with the tumor proliferation index (% of Ki67 positive cells). The highest tumor proliferation index was detected in the tumor + placebo group (Figure 5). Both CTx groups had a significantly lower tumor proliferation index when compared with both groups that did not receive CTx.

**Figure 6:**
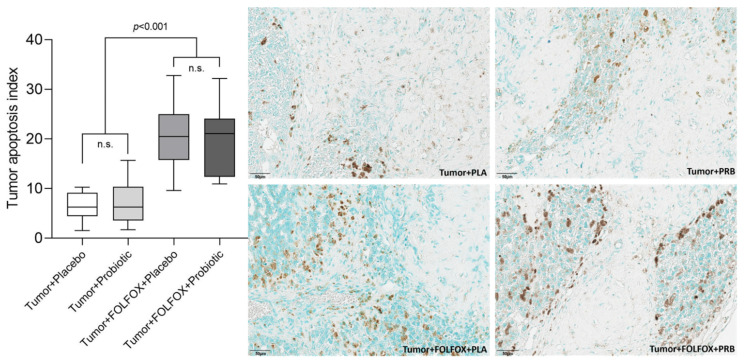
Tumor apoptosis index (% of TUNEL+ cells in the tumor). Values outlying ± 2SD range excluded from analysis. n.s.—not significant.

A significantly higher tumor apoptosis index (% of TUNEL positive cells) was seen in samples from rats that received CTx (Figure 6). We did not observe any tumor apoptosis index differences between rat groups that did not receive CTx.

**Figure 7:**
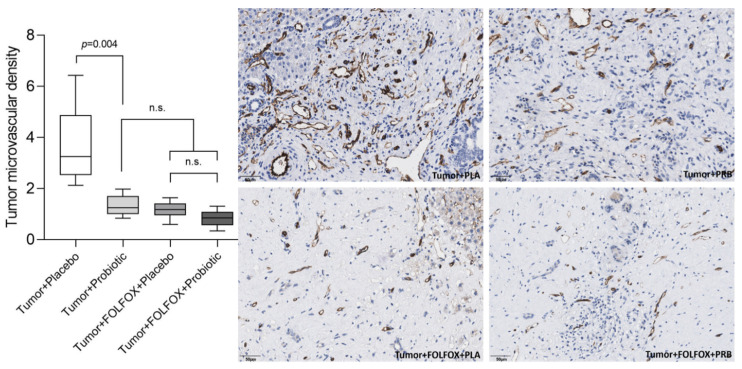
Tumor microvascular density (number of vessels sprouts per 1000 µm^2^). Values outlying ± 2SD range excluded from analysis. n.s.—not significant.

Probiotic supplementation alone, without FOLFOX therapy, significantly reduced tumor microvascular density (MVD) (Figure 7). MVD differences were significant between both rat groups that did not receive CTx. Furthermore, there was no significant difference between the tumor + probiotic group and both rat groups that received FOLFOX CTx.

## 3. Discussion

CRCLM is one of the major causes of death for CRC, with a reported five-year survival rate of less than 20% [8]. Long-term cure involves complete surgical removal of the metastatic lesions, however, only a small number of patients are candidates for such treatment option upon diagnosis. In these cases, novel treatment additives are crucial for controlling the disease and prolonging patient survival.

Over the last 20 years the use of probiotics as a tool in cancer prevention and therapy gained a lot of research attention [19]. Most of the current publications analyze local probiotics effects in reducing local CRC growth and alleviating CTx induced intestinal mucositis with its complications [19,20]. Only a handful of available studies investigate the general tumor-suppressing response initiated by probiotic supplementation. Unfortunately, none of the latter research look into the great problem of CRC metastasis. Therefore, the aim of our study was to evaluate probiotic supplementation antitumorigenic effects in a CRCLM rat model.

This study shows the effects of multispecies probiotic supplementation on CRCLM growth. One of the most important findings is that probiotic supplementation has a significant tumor-growth-suppressing effect. Our IHC analysis of the tumor samples indicates that the tumor-growth suppression was mainly caused by reducing tumor microvasculature. Angiogenesis is crucial during tumor development as the supply of oxygen and nutrients is necessary to sustain continuous growth and obtain metastatic potential [21]. To date, only a handful of studies have analyzed probiotic impact on tumor growth in vivo but none of them did it in an orthotopic liver tumor model [22,23,24,25,26].

The exact probiotic acting mechanisms are unknown, but a few studies suggested several possible pathways. A study by Li et al. investigated the role of a specific probiotic mixture on hepatocellular carcinoma growth [24]. Tumor growth was significantly reduced in the probiotic supplementation group when compared to controls. After performing an extensive tumor analysis and investigation of the overall probiotic caused immunological changes, the authors determined that the probiotic mixture down-regulated IL-17 by decreasing the Th17 cell count and, thus, resulted in decreased tumor microvasculature. A study by Aindelis et al. also managed to reduce tumor growth by administering mice with *Lactobacillus casei* [25]. They concluded that this probiotic was responsible for increased immune response and enhanced tumor infiltration by CD^8+^ cells. Additionally, a very similar probiotic immunomodulatory effect, resulting in increased numbers of CD^8+^ cells in the tumor, was reported by Shang et al. [23]. A study by Chen et al. concluded that a probiotic consisting of *Clostridium butyricum* reduces colorectal cancer growth in the intestine [26]. Their analysis showed that *C. butyricum* modulated Wnt/β-catenin signaling and the gut microbiota. Summing up the pathways, different probiotics may have a lot of different tumor-suppressing effects, immunomodulation being one of the most important [27,28].

As the results of TUNEL staining indicates that probiotic supplementation mainly exerted tumor-growth inhibition but did not have any cytotoxic effect. The tumor proliferation index was lower in rats receiving probiotics, but the difference, when compared to the placebo, did not reach statistical significance. These findings indicate that probiotics combined with FOLFOX CTx may have a synergistic effect as they act through different pathways, one inhibiting angiogenesis and the other being cytotoxic, although we could not prove this in our study in terms of tumor size; further research examining this theory is of interest.

For several years our study group has been investigating different cancer-suppressing agents in an orthotopic CRCLM rat model. We have previously determined that both glycine and melatonin substantially suppress CRCLM growth in vivo [29,30]. Currently, we are conducting a similar study looking into possible combinations of these novel tumor-suppressing agents and their possible synergistic effects. To this date, there are no published clinical studies investigating the effects of probiotics on CRC tumor growth. According to the Clinicaltrials.gov (accessed on 14 May 2022) database, a study examining VSL#3 probiotic supplementation in increasing CRC major response rate is recruiting patients and their findings are eagerly awaited (ClinicalTrials.gov Identifier: NCT01579591).

It should be noted that this is an animal study, thus, it is theoretically possible that probiotic supplementation could have a different interaction with the human immune system, leading to different anti-tumorigenic outcomes. Furthermore, as Lazaris et al. note in their article, liver metastasis have different histopathological growth patterns and, unfortunately, animal models do not fully represent the diversity found in human livers [31].

## 4. Materials and Methods

### 4.1. Animals

Six-week-old male Wistar rats (Janvier Labs, Le Genest-Saint-Isle, France) weighing approximately 200–280 g at the start of the experiment were used. Animals were housed at the Institute for Biomedical Research (Medical University of Graz, Graz, Austria). Two to four rats were housed per cage with unrestricted access to pelleted chow and tap water and kept under controlled conditions of temperature (22–23 °C) and humidity (45–50%) in a 12 h light/dark cycle. All procedures were approved by the Austrian Committee for Animal Trials (Approval number: BMWF-66.010/0158-V/3b/2019) and performed according to the 3R guidelines.

### 4.2. Experimental Groups and Study Design

**Figure 8:**
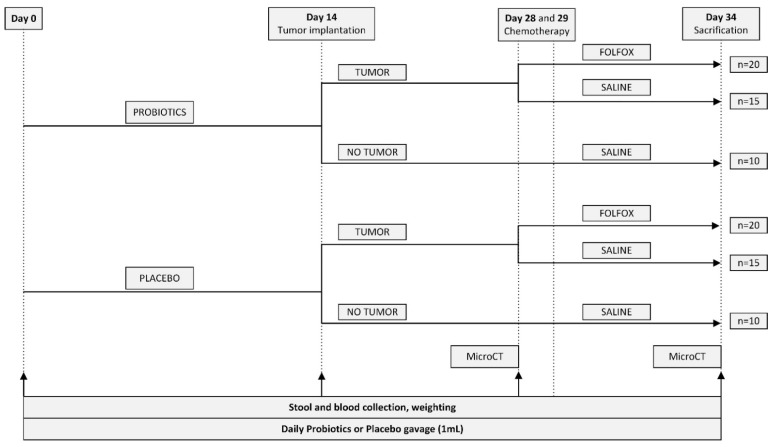
Experimental design of the study.

All rats were randomly assigned to six experimental groups (Table 1). The overall length of the protocol lasted 34 days. For the first 14 days, rats received daily probiotics or placebo gavage. On day 14 rats underwent tumor implantation or sham surgery. FOLFOX CTx or saline was administered on experimental days 28 and 29 after the first micro-CT scan was performed. On day 34 after performing the second micro-CT scan rats were euthanized and tissue samples were collected. The detailed scheme of the experimental design is presented in Figure 8.

### 4.3. Probiotics

A commercially available multispecies probiotic mixture (provided by Institut Allergosan, Graz, Austria) comprising eight bacterial strains was used. *Lactobacillus casei W56; Lactobacillus acidophilus W37; Lactobacillus brevis W63; Lactococcus lactis W58; Bifidobacterium lactis W52; Lactococcus lactis W19; Lactobacillus salivarius W24*, and *Bifidobacterium bifidum W23* were included in 1 g of matrix (maize starch, maltodextrins, vegetable protein, potassium chloride, magnesium sulphate, amylases, and manganese sulphate). The matrix without bacteria was used as placebo. The probiotic/placebo powder was dissolved using tap water every morning approximately 15 min before gavaging. Each rat received 1 mL of suspension containing either 1.2 × 10^9^ CFU/mL of the probiotic or the same amount of microbe-free placebo mixture.

### 4.4. Tumor Implantation

**Figure 9:**
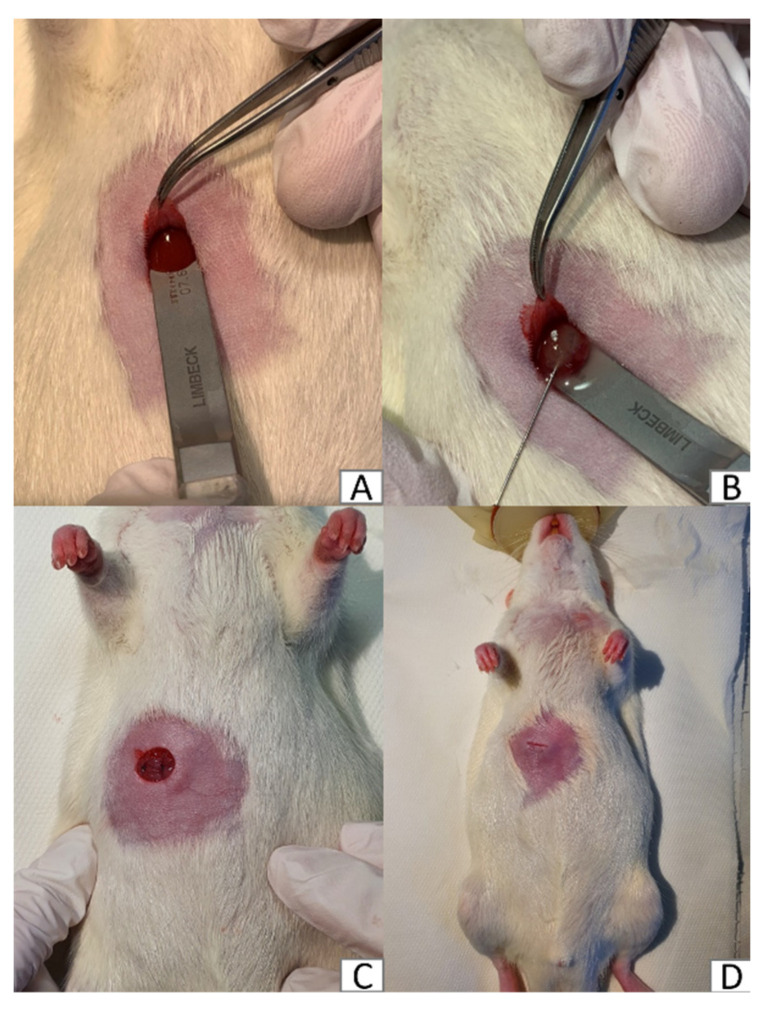
Colorectal adenocarcinoma tumor implantation. (**A**) exposure of the median liver lobe. (**B**) Tumor inoculation. (**C**) Wound closure using a double layer interrupted suture. (**D**) Skin adapted using topical adhesive.

The rat colorectal cancer cell line (CC531) (Cell Lines Service, Eppelheim, Germany) was cultured in RPMI-1640 medium (Gibco, Thermo Fisher Scientific, Waltham, MA, USA) supplemented with 10% fetal bovine serum (Global Life Sciences Solutions, Marlborough, MA, USA), 1% Penicillin/Streptomycin (Sigma-Aldrich, St. Louis, MO, USA), 1% L-glutamine (Gibco, Thermo Fisher Scientific, Waltham, MA, USA), and 25 mM HEPES (Gibco, Thermo Fisher Scientific, Waltham, MA, USA) at 37 °C in a humidified atmosphere containing 5% CO_2_. On the morning of tumor implantation CC531 cells were harvested and suspended at a density of 5 × 10^6^ cells in 100 µL of sterile phosphate-buffered saline (PBS). The tumor inoculation procedure was performed under general anesthesia (with 2% isoflurane) with additional intramuscular injection of fentanyl (5 µg/kg). After induction of anesthesia, the animal was placed in a supine position. The right subcostal area was shaved and disinfected. Afterwards, a 1 cm subcostal incision approximately 0.5 cm below the ribcage was made. After reaching the peritoneum the median liver lobe was gently exposed using the rear end of microsurgical forceps (Figure 9A). Using a 25-Gauge needle 100 µL of the prepared CC531 cell suspension or 100 µL of sterile PBS for sham surgery was slowly injected under the liver capsule (Figure 9B). An appearance of a whitish protrusion indicated successful tumor implantation. A two-layer interrupted suture (Vicryl 4-0, Ethicon, Somerville, NJ, USA) was performed to close the abdominal wound (Figure 9C), additionally, the skin was adapted and glued with topical skin adhesive (LiquiBand Optima, Advanced Medical Solutions, Devon, UK) (Figure 9D). At the end of the operation, all animals received 4.5 mg/kg carprofen subcutaneously. To further reduce pain drinking water was supplemented with ibuprofen (0.2 mg/mL H_2_O) for the first five postoperative days.

### 4.5. Micro-CT Scanning and Image Analysis

24 h before the first Micro-CT scan a single dose (800 µL) of ExiTron nano 12,000 (Viscover, nanoPET Pharma GmbH, Berlin, Germany) contrast agent was injected via the tail vein. In this study, we used the SkyScan 1276 (Bruker, Karlsruhe, Germany) micro-CT scanner using the following scanning parameters: 1 mm filter, 85 kV, 200 µA, exposure time 132 ms, detector binning 4 × 4, and resolution of 40.5 µm. Anesthesia induction was performed using 5% isoflurane, afterwards, the rat was transferred to the rat cassette. Anesthesia was maintained using 3% isoflurane with continuous heart and respiratory rate monitoring.

The micro-CT image analysis was performed using CTAn software (Bruker, Karlsruhe, Germany) by a single investigator. At first, the reconstructed images were filtered, then the liver volume was calculated by running a prespecified task list. The tumor volume was measured by first defining a custom region of interest based on the tumor shape and size and then running a prespecified task list. Change in tumor volume was expressed as tumor volume at day 28 (mm^3^) × 100%/tumor volume at day 34 (mm^3^).

### 4.6. Chemotherpy

The FOLFOX CTx regimen was chosen in this experiment due to its proven potency in treating CRCLM [32,33]. This regimen was also used in another similar study by our research group with the desired tumor suppressive effect [29,30]. On day 28 a mixture of 100 mg/m^2^ Calcium folinate (Sandoz, Holzkirchen, Germany), 42.5 mg/m^2^ Oxaliplatin (Fresenius Kabi, Bad Homburg, Germany) and 500 mg/m^2^ 5-Fluoruracil (Sandoz, Holzkirchen, Germany) was used. Twenty-four hours after the first dose, the second dose of 100 mg/m^2^ Calcium folinate and 500 mg/m^2^ 5-Fluoruracil was injected. The doses were calculated according to the animals’ skin surface as described elsewhere [34]. All CTx agents were administered via intraperitoneal injections under short general 2% isoflurane anesthesia.

### 4.7. Blood Sample Analysis

On experimental days 0, 14, and 28, venous blood samples were collected from the subclavian vein under 2% isoflurane anesthesia. On the last experimental day, blood was collected from the inferior vena cava during terminal blood drawing. Complete blood count was measured using a V-Sight hematology analyzer (A. Menarini Pharma GmbH, Vienna, Austria).

### 4.8. Immunohistochemical Staining

Tissue samples were fixed in 4% buffered formaldehyde solution, washed with distilled water, and dehydrated with ascending ethanol series. Following incubation at 60 °C, tissues were embedded in paraffin. 2 μm thick tissue sections were cut using a rotary microtome.

Immunohistochemical staining was performed using the following primary antibodies: Anti-MPO (Dako, Via Real Carpinteria, CA, USA, A0398, dilution 1:800), Anti-Ki67 (Abcam, Cambridge, UK; ab16777, dilution 1:200), and Anti-CD31 (Abcam, Cambridge, UK; ab182981, dilution 1:2000). The UltraVision LP Detection System: HRP Polymer (Thermo Fisher Scientific, Waltham, MA, USA) and DAB Chromogen (Dako, Via Real Carpinteria, CA, USA) were used to visualize the target antigen. Sections were counterstained with hematoxylin. Liver apoptotic cells were assessed by the terminal deoxynucleotidyl transferase-mediated dUTP nick-end labelling (TUNEL) technique using a TUNEL Assay Kit (Abcam, Cambridge, UK; ab206386) according to the manufacturer’s manual.

MVD was calculated as the number of vessel sprouts per 1000 µm^2^ of tumor area.

All stained sections were scanned and positive cells were counted from the tumor area using the open source QuPath software [35]. Tumor microvasculature quantification analysis was performed additionally using the ImageJ software (U.S. National Institutes of Health, Bethesda, MD, USA).

### 4.9. Statistical Analysis

Analysis was performed using SPSS 23.0 (IBM Corp., Armonk, NY, USA) and GraphPad Prism 9 (GraphPad Software, La Jolla, CA, USA). A *p*-value of less than 0.05 was considered significant. Distribution of the variables was investigated using the Shapiro–Wilk test. As the data was not normally distributed, continuous variables were analyzed using Kruskal–Wallis with Dunn’s post-hoc test. Data is reported as median and quartiles (Q1; Q3).

## 5. Conclusions

This study shows that a multispecies probiotic mixture (1.2 × 10^9^ CFU/daily) alone, without addition of FOLFOX CTx, significantly reduces angiogenesis and inhibits colorectal cancer liver metastasis growth in an experimental CRCLM model. Further investigations are warranted to explain the exact bacteria promoting this effect and the underlying molecular mechanisms.

## Figures and Tables

**Table 1:** Experimental animal groups.

Gavage	CONTROL Tumor (−)/FOLFOX (−)		Non-FOLFOX Tumor (+)/FOLFOX (−)		FOLFOX Tumor (+)/FOLFOX (+)	
	Placebo	Probiotics	Placebo	Probiotics	Placebo	Probiotics
Started (n)	10	10	15	15	20	20
Finished (n)	10	10	15	15	20	19 *

* One death on the last protocol day due to chemotherapy toxicity.

## Data Availability

All data relevant to the study are included in the article.
